# Setting Yourself up for Success as an Early Career Investigator in Clinical Trials

**DOI:** 10.1016/j.jacadv.2026.102763

**Published:** 2026-04-25

**Authors:** Aditya S. Bharadwaj, Nosheen Reza, Newton Wiggins, Jennifer Rymer, F. Aaysha Cader, Roxana Mehran

**Affiliations:** a Division of Cardiology, Leonard M. Miller School of Medicine, University of Miami, Miami, Florida, USA; b Division of Cardiovascular Medicine, Department of Medicine, Perelman School of Medicine at the University of Pennsylvania, Philadelphia, Pennsylvania, USA; c Division of Cardiology, The Chattanooga Heart Institute, Chattanooga, Tennessee, USA; d Division of Cardiology, Duke University Medical Center, Durham, North Carolina, USA; e Department of Cardiology, Kettering General Hospital NHS Foundation Trust, Kettering, United Kingdom; f Mount Sinai Fuster Heart Hospital, New York, New York, USA

**Keywords:** clinical trials, early career, principal investigator

**Figure:**
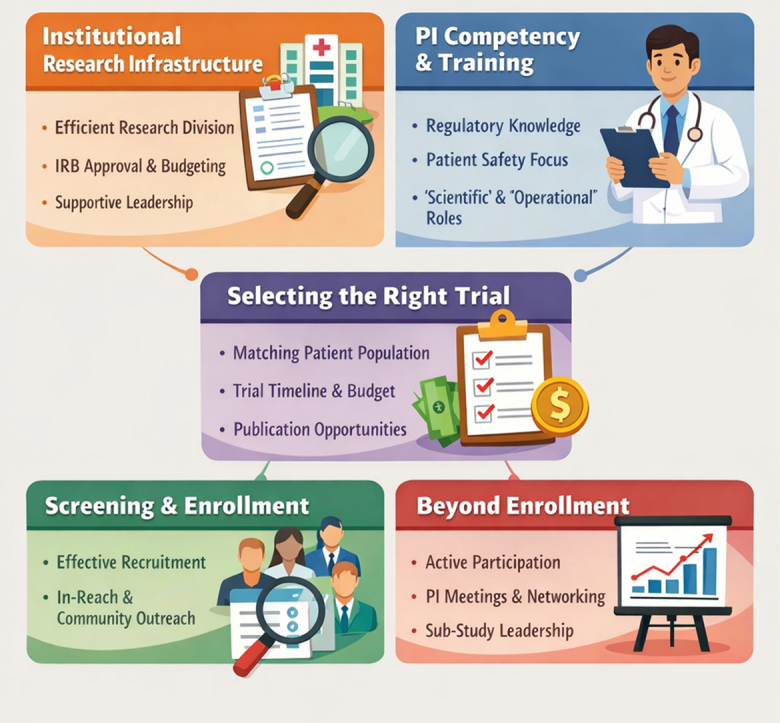

Randomized controlled trials (RCTs) are crucial for generating evidence for management of patients. This is particularly true for cardiology, wherein a rapidly evolving pharmacotherapeutic and devices landscape warrants the conduct of large well-designed, pragmatic trials. The results from RCTs often have significant implications on societal guidelines, regulatory decisions, and ultimately patient care. Completion of a successful RCT requires the commitment of principal investigators (PI) who will set up sites, and appropriately delegate teams to screen, randomize, and provide the necessary follow-up of patients.^1^ Early career (EC) cardiologists have a significant opportunity to participate in these trials as PIs, thereby further progressing their research careers. The involvement of EC cardiologists in clinical trials is also a systematic necessity to create a strong pipeline of future trialists. Previous publications have addressed the challenges for “scientist researchers” in terms of securing federal grants and funding for other investigator-initiated research projects, with over U.S. $1.8 billion of National Institutes of Health grant funding being recently terminated.^2^^,^^3^ In addition, the clinical workload, limited protected research time, and lack of academic recognition also pose potential challenges to EC cardiologists who are interested in pursuing research. There is also a paucity of literature in terms of guidance for “clinician scientists” or “pure clinicians” with limited experience in research conduct,^4^ who wish to be involved as site PIs in clinical trials.

In this paper, we provide an outline and framework for successful involvement in RCTs for site PIs who may be in their early or mid-career stages. We identified the following key components to be successful as an investigator (Figure 1). Although this is predominantly a U.S.-based perspective, the authors believe that the key components have the potential for global application.

1. Institutional research infrastructure: An efficient and adequately staffed research division is a prerequisite for success in a clinical trial. Well-trained research coordinators with in-depth knowledge of regulatory processes and research best practices are essential. However, oversight of coordinator’s work including regulatory paperwork, accuracy of data entry, answering queries from study sponsor, and timely completion of these tasks, is the responsibility of the study PI. New study start-up at an institution often involves obtaining a nondisclosure agreement, approval by internal research committee, budget negotiation, legal contracting, and Institutional Review Board approval culminating with site initiation. It is important to have an efficient workflow to expedite these processes so that the site does not “lose out” on time for enrollment. It is important for an EC interviewing for new job positions to do a deep dive into the current state of research affairs at the institution, support from leadership and potential allocation of dedicated time, if they plan to pursue clinical research.
2. Investigator competency and training: The study PI is ultimately responsible for the integrity and ethical conduct of the study and should be well versed with the codes of conduct outlined by the U.S. Food and Drug Administration for protecting the rights, safety, and welfare of study subjects.^5^ PI responsibilities range from ensuring strict adherence to study protocol, obtaining informed consent, ensuring secure maintenance of data and records with the help of study coordinator, and timely reporting of adverse events, ensuring Institutional Review Board compliance and disclosure of all financial conflicts of interest.^6^ For those EC PIs looking for a formal didactic, the American College of Cardiology’s Clinical Trial Research Program provides training and networking opportunities for young aspiring clinical trialists, while fostering diversity and inclusion in research workforce.^7^ In the United Kingdom, the National Institute of Health and Care Research Associate PI program offers 6-months training opportunities along with mentorship.^8^ Pursuing a postgraduate degree in Clinical Research can also be an invaluable resource. The ability to understand these nuances of a clinical trial may help EC PIs implement designs to facilitate faster and more efficient enrollment, while also enabling the inclusion of more diverse and traditionally under-represented patient populations, who are often less likely to participate in conventional trials.
3. Picking the “right” clinical trial: PIs should pick clinical trials where they have the maximum opportunity to make a significant contribution to the trial. This includes making sure they have the “right” patient population which meets the inclusion criteria for the clinical trial. It is also important to assess the overall timeline of the trial including the percentage of patients who have already been enrolled thus far and take into consideration the time needed at their own institution for study start-up. It is also prudent to consider the finances of the study including a breakup of the dollar amount paid by the sponsor for enrollment, follow-up, PI, and coordinator fees. It is also reasonable to ascertain what opportunities may exist if the PI manages successful recruitment to be named in later publications.
4. Screening and enrollment: Screening and enrollment often require in-reach within the institution as well as community outreach with dissemination of information regarding the clinical trial and the inclusion criteria. Site PIs can boost their chances of authorship in the main clinical trial publication because high enrollment and data completion are 2 important metrics that are taken into account during authorship assignments in multicenter trials.^9^ It is important for the site PI to have buy-in from their sub-PIs, subspecialty colleagues, and sometimes other specialties as well.
5. PI involvement beyond enrollment: Site PIs who are looking to maximize their contribution should stay involved with the clinical trial, beyond just enrolling patients. These include attending PI meetings, volunteering to present cases and contributing to discussions during these meetings. Networking during such PI meetings and demonstration of commitment to research may pave way for opportunities in newer clinical trials. They are also useful learning opportunities for open peer-to-peer discussion on methods to improve recruitment and experience-sharing on trouble-shooting issues during the trial. PIs may also use the opportunity to learn from senior trialists regarding data analysis, interpretation, and the overall methodology for running RCTs, because there is often a lack of formal didactic to learn these. PIs may also proactively seek opportunities for leading substudies and writing manuscripts.

**Figure 1:**
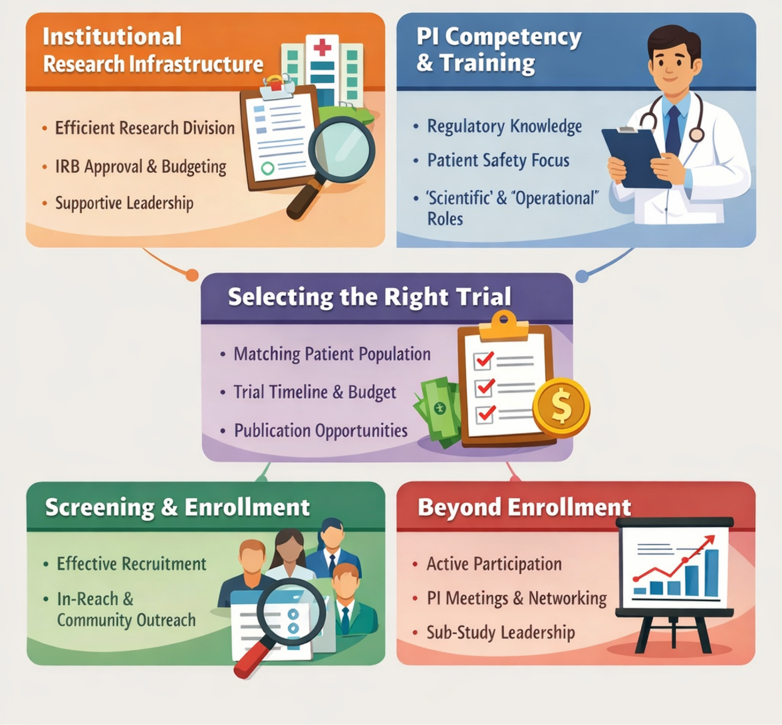
**Key Components for Success as a Clinical Trial Investigator** IRB = Institutional Review Board; PI = principal investigator.

In summary, successful involvement as a site PI in RCTs provides a significant opportunity to EC cardiologists to advance their research careers. A supportive institutional infrastructure, in-depth knowledge of research methodology, picking the right clinical trial, and maximizing enrollment are prerequisites for success.

## Funding support and author disclosures

Dr Reza is supported by the 10.13039/100000050National Heart, Lung, And Blood Institute of the National Institutes of Health under Award Number K23HL166961. Dr Rymer is supported by the 10.13039/100000050National Heart, Lung, And Blood Institute of the National Institutes of Health under Award Number 1UG3HL171357. Dr Bharadwaj is a speaker and consultant for 10.13039/100020297Abiomed, Shockwave Medical Cardiovascular Systems Inc (CSI) and he has received honoraria for the same. Dr Reza has received consulting/speaking honoraria from Zoll, Inc., 10.13039/100016545Roche Diagnostics, 10.13039/100016473American Regent, Bristol Myers Squibb, 10.13039/100004325AstraZeneca, 10.13039/501100016198Idorsia, and 10.13039/501100004191Novo Nordisk; and has received research grants to the institution from Bristol Myers Squibb. Dr Rymer is a speaker and consultant for 10.13039/100020297Abiomed, Viatris, Bristol Myers Squibb, 10.13039/501100004191Novo Nordisk, and 10.13039/100000046Abbott. All other authors have reported that they have no relationships relevant to the contents of this paper to disclose.
